# Supplementary material for: Lignocellulose-Degrading Enzymes: A Biotechnology Platform for Ferulic Acid Production from Agro-Industrial Side Streams
Source: Foods. 2021 Dec 8;10(12):3056. doi: 10.3390/foods10123056 (PMC8701750; doi:10.3390/foods10123056)
Supplement: Supplementary file 1 [file foods-10-03056-s001.zip › foods-1473962-supplementary.pdf]

Supplementary Materials

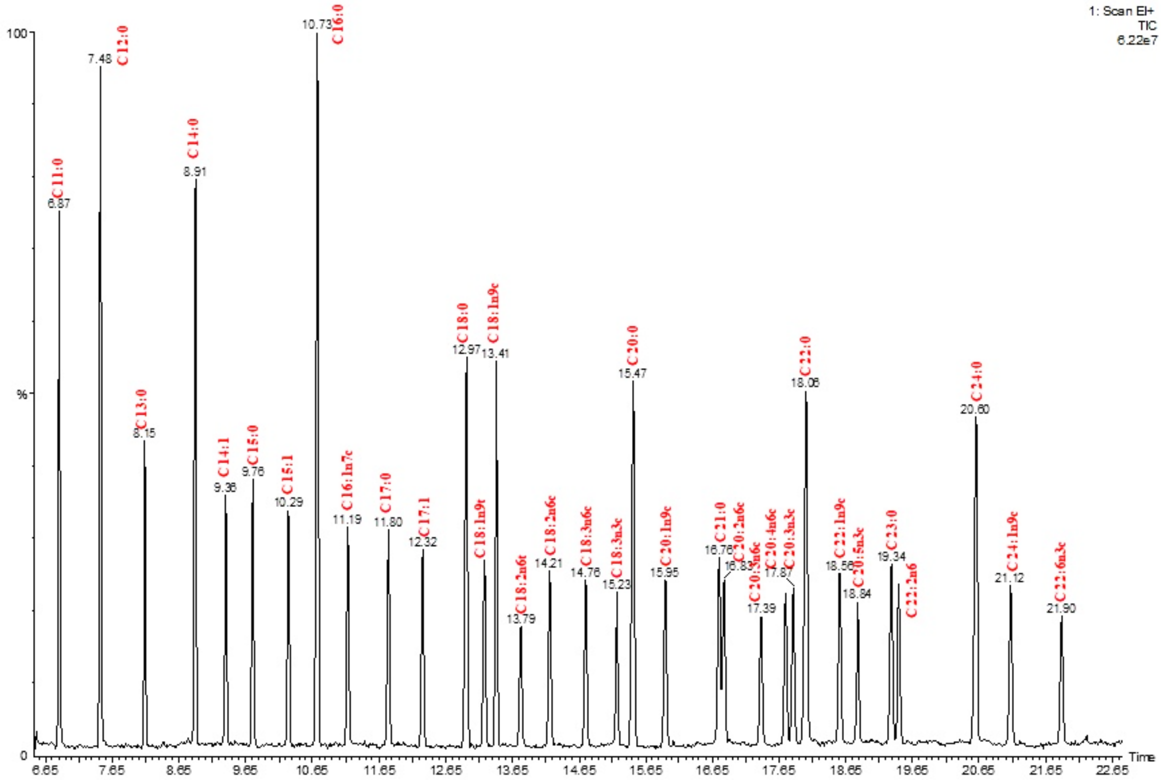

Figure S1. Chromatographic separation of FAMEs profile of C_4-C_{24} reference material using GC/MS: 6.87 (C11:0 – undecanoic acid), 7.48 min (C12:0 – lauric acid), 8.15 min (C13:0 – tridecanoic acid), 8.91 min (C14:0 – myristic acid), 9.36 min (C14:1– myristoleic acid), 9.76 min (C15:0 – pentadecanoic acid), 10.29 min (C15:1 – *cis*-10-pentadecanoic acid), 10.73 min (C16:0 – palmitic acid), 11.19 min (C16:1n7c – palmitoleic acid), 11.80 min (C17:0 – heptadecanoic acid), 12.32 min (C17:1 – heptadecenoic acid), 12.97 min (C18:0 – stearic acid), 13.24 min (C18:1n9t – elaidic acid), 13.41 min (C18:1n9c – oleic acid), 14.21 min (C18:2n6c – linoleic acid), 14.76 min (C18:3n6c – γ-linolenic acid), 15.23 min (C18:3n3c – linolenic acid), 15.95 min (C20:1n9c – eicosenoic acid), 17.76 min (C20:4n6c – arachidonic acid), 18.06 min (C20:3n3c – docosanoic acid), 18.56 min (C22:1n9c – erucic acid), 18.84 min (C20:5n3c – eicosapentaenoic acid), 19.34 min (C23:0 – tricosanoic acid), 19.46 min (C22:2n6c – docosadienoic acid), 20.60 min (C24:0 – lignoceric acid), 21.12 min (C24:1n9c – nervonic acid), 21.90 min (C22:6n3c – docosahexaenoic acid).

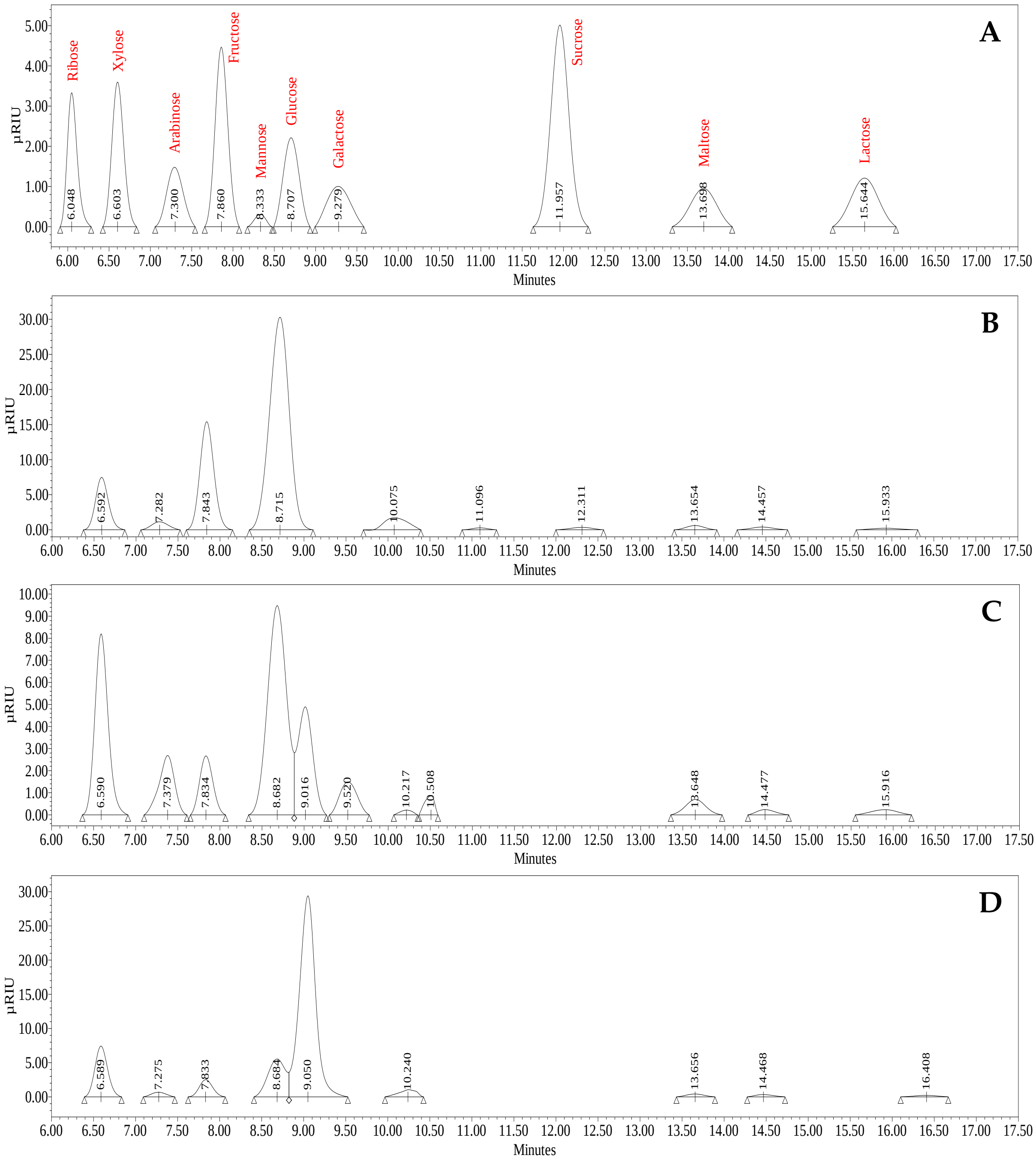

Figure S2. HPLC-RID chromatographic separation of mono- and disaccharides in a mixture of standards (A) bran hydrolysates obtained after EH with Viscozyme L (B), Viscoferm (C), and Celluclast 1.5 L (D).
